# FHQ-RRT*: An Improved Path Planning Algorithm for Mobile Robots to Acquire High-Quality Paths Faster

**DOI:** 10.3390/s25072189

**Published:** 2025-03-30

**Authors:** Xingxiang Dong, Yujun Wang, Can Fang, Kemeng Ran, Guohui Liu

**Affiliations:** College of Computer and Information Science, Southwest University, Chongqing 400715, China; d13538@email.swu.edu.cn (X.D.); wangyjun@swu.edu.cn (Y.W.); rkm666@email.swu.edu.cn (K.R.); liu914904@email.swu.edu.cn (G.L.)

**Keywords:** path planning, sampling-based algorithms, rapidly explored random tree, optimal path planning

## Abstract

The Rapidly-exploring Random Tree Star (RRT*) algorithm, widely utilized for path planning, faces challenges, such as slow acquisition of feasible paths and high path costs. To address this issue, this paper presents an improved algorithm based on RRT* that can obtain high-quality paths faster, termed Faster High-Quality RRT*(FHQ-RRT*). The proposed algorithm enhances the exploration efficiency and path quality of mobile robots through three key innovations: First, a dynamic sparse sampling strategy that adaptively adjusts the sampling density according to the growth rate of the random tree, thereby increasing the algorithm’s growth speed while maintaining adaptability to complex environments. Second, a new node creation method that combines the bisection method, triangle inequality, and the concept of KeyPoints to reduce the cost of creating new nodes. Third, a focused rewiring strategy that restricts the rewiring operation to valuable regions, thereby improving rewiring efficiency. The performance of FHQ-RRT* was validated in four simulation maps and compared with other algorithms. In all validated maps, FHQ-RRT* consistently achieved the lowest path cost. Regarding time cost, FHQ-RRT* reduced the planning time by over 40% in the circular-obstacle map, 77% in the simple maze map, 56% in the complex maze map, and 50% in the narrow map. The simulation results show that FHQ-RRT* can rapidly generate high-quality paths faster than other algorithms.

## 1. Introduction

In the context of today’s rapid development of intelligent and automation technologies, path planning algorithms, as core technologies in the fields of autonomous navigation, intelligent transportation, and robotic systems, are gradually becoming the core focus of research in multiple fields. The main task of path planning algorithms is to generate feasible paths from the starting point to the end point for various types of mobile entities (e.g., robots, self-driving vehicles, and unmanned aerial vehicles) in complex and changing environments while meeting the key requirements of safety, high efficiency, and dynamic adaptability. Currently, path planning algorithms are widely used in many fields, including driverless vehicles [1,2], additive manufacturing [3,4], and biology [5]. With the increasing complexity of application scenarios, the performance of path planning algorithms must also improve.

A significant amount of research has been devoted to the path planning problem. Commonly used path planning methods include artificial potential field-based methods [6], geometric-based methods [7], learning-based methods [8,9], and sampling-based methods [10,11]. However, each of these methods has inherent limitations. Field-based potential artificial methods may encounter issues with local minima in complex environments, leading to inaccurate path planning. Geometric-based methods, such as A* [12] and Dijkstra [13], perform poorly in complex and high-dimensional spaces with limited processing capabilities. Improved A* [14] optimizes the path planning problem of the A* algorithm in large scenes by leveraging the connectivity relationships of nodes in the topological map, thereby enhancing the planning efficiency of the algorithm. Dynamic A* [15] incorporates a distance constraint and dynamically selects checkpoints to replan the path, thereby enabling the mobile robot to dynamically plan a feasible path to the destination within a limited distance while approaching the shortest path as closely as possible. Learning-based methods require large amounts of training data and computational resources, which can significantly limit their practical application. Intelligent optimization algorithms include Genetic Algorithms [16,17], Particle Swarm Optimization [18], Ant Colony Algorithms [19], and path planning algorithms based on reinforcement learning. The Adaptive Genetic Algorithm [20] is an improvement over the basic genetic algorithm, which greatly improves the convergence accuracy and accelerates the convergence speed.

In contrast, sampling-based methods offer broad applicability and high search efficiency. They can quickly find feasible paths, even in high-dimensional spaces, and perform exceptionally well in large environments. These advantages render sampling-based algorithms particularly prominent in various application scenarios. Rapid-exploring Random Tree (RRT) [21] is a typical representative of sampling-based algorithms and is known for its ability to quickly generate feasible paths. Rapid-exploring Random Tree Star (RRT*) [22] represents a milestone variant of the RRT algorithm. It introduces the ChooseParent and Rewire processes on the basis of the RRT. In the ChooseParent phase, RRT* selects a parent node from the neighbors of the new node, and the Rewire process optimizes and rewires the path, providing probabilistic completeness and asymptotic optimality. Although RRT* theoretically guarantees an optimal solution, its slow convergence speed makes it challenging to compute the optimal path within a finite time. Numerous variants of RRT* have been developed to address this issue. Ran et al. proposed a path planning algorithm based on the Rapidly-exploring Random Tree Star (RRT*) algorithm [23] for mobile robots under kinematic constraints. An improved RRT*(IBPF-RRT*) [24] guides the random tree towards the target point by optimizing the sampling part to enhance planning speed and proposes a path optimization strategy to reduce redundant inflection points and path cost. Goal-oriented RRT* (GRRT*) [25] and Dynamic Environment RRT* (ED-RRT*) [26] reduce the sampling process time through goal-oriented sampling strategies. However, both methods suffer from local optima. The Fast-RRT* [27] algorithm employs a Hybridsampling strategy that combines goal-oriented sampling with constraint sampling during the Sampling phase, which reduces the ambiguity of the sampling process. However, this algorithm performs poorly in concave-map environments. S. Huang [28] proposed an improved algorithm that integrates the artificial potential field method with the Rapidly-exploring Random Tree algorithm. This approach utilizes the gravitational and repulsive fields of the artificial potential field to reduce the randomness inherent in the path planning process, thereby enhancing the search efficiency of the RRT algorithm. Q. Zhang [29] proposed a novel hybrid path planning algorithm based on the Rapidly-exploring Random Tree algorithm. By incorporating heuristic search principles into the random extension process of RRT, the algorithm is able to plan more efficient paths in a reduced time frame. H. Yang [30]. proposed an improved RRT-Connect algorithm that incorporates a target bias strategy into the traditional RRT-Connect framework. This enhancement allows the algorithm to outperform conventional methods in terms of both path length and execution time. However, despite these improvements that enhance the traditional RRT algorithm and demonstrate its superiority, some disadvantages and limitations remain, particularly in scenarios involving multiple obstacles or dynamic environments. RRT*-Normal (RRT*N) [31] was proposed to improve the speed of finding the target path, which generates new nodes through a probability distribution such that nodes close to the target have a higher probability. Faster RRT* (F-RRT*) [32] introduced the concept of creating parent nodes for the sampling points. The ChooseParent phase incorporates the FindReachest and CreateNode processes to generate new nodes as parent nodes for the sampling points to improve the path quality. However, the high cost associated with creating new nodes and the issue of creating redundant nodes hinder the speed of the algorithm in obtaining an initial solution. The Fast Forward-RRT* (FF-RRT*) [33] algorithm improves upon the Hybridsampling strategy of Fast-RRT* and addresses its application limitations. In the ChooseParent phase, FF-RRT* fully inherits its strategy from F-RRT*. The performance benefits obtained from the enhanced sampling strategy offset the performance associated with the ChooseParent phase. However, FF-RRT* cannot resolve the fundamental shortcomings of F-RRT*. More Quickly-RRT* (MQ-RRT*) [34] introduces a new hybrid sampling strategy that combines a sparse sampling mechanism with a dynamic goal-biased strategy. This approach provides guidance during the sampling process while avoiding repeated sampling in the same space. MQ-RRT* also proposes a method for creating new nodes and improving path quality in environments with circular obstacles. However, the algorithm performs poorly in narrow spaces.

In the field of path planning, sensor technology plays a crucial role. Sensors can provide mobile robots with environmental information, including the location, shape of obstacles, and dynamic changes in the environment, thereby providing necessary input data for path planning algorithms. During the path planning process, the acquisition and processing of sensor data are crucial steps. Typically, mobile robots are equipped with various types of sensors, such as LiDAR, cameras, and ultrasonic sensors. These sensors can obtain environmental information in real time and convert it into data that can be used for path planning. For example, LiDAR can provide high-precision environmental distance information, while cameras can capture visual information of the environment to identify the shape and texture of obstacles. In this paper, the proposed algorithm assumes that sensor data has been preprocessed to extract obstacle and free space information from the environment, which is then used as input for the path planning algorithm. More attention is focused on the path planning itself.

Inspired by the aforementioned algorithms, a new path planning algorithm, FHQ-RRT*, is proposed on the basis of F-RRT* and MQ-RRT*. It can acquire high-quality paths at a faster speed and solve the pathfinding failure issue of MQ-RRT* in narrow-map environments. The main contributions of this paper are as follows:

1. Dynamic Sparse Sampling Strategy: A strategy is proposed that dynamically adjusts the exploration speed and precision of the algorithm on the basis of the growth rate of the random tree, enhancing the adaptability of the algorithm to the environment.

2. New Node Creation Method and KeyPoints Concept: The new algorithm actively creates parent nodes for the sampling points through dichotomy and collision detection instead of choosing from existing nodes. The newly created parent nodes are closer to obstacles and can effectively reduce the path cost. All newly created parent nodes are defined as KeyPoints, and no new parent nodes must be created when the temporary parent node of a new sampling point is a key point. This improvement reduced the number of parent node creations and increased the efficiency of the algorithm.

3. New Rewire Strategy: A strategy that focuses the rewire operation on more valuable nodes, thereby improving the efficiency of rewiring.

The rest of this paper is organized as follows. Section 2 presents the necessary background for this paper, including the relevant definitions of path planning and the principles of reference algorithms. The proposed FHQ-RRT* method is introduced in Section 3. Section 4, and Section 5 describes the simulation environment and results with a comparative analysis of RRT*, F-RRT*, and MQ-RRT*. Section 6 concludes the paper and presents possible future research directions.

## 2. Preliminaries

This section formalizes the motion planning problem and introduces the F-RRT* and MQ-RRT* algorithms, which inspire the proposal of the FHQ-RRT* algorithm.

### 2.1. Problem Definition

Let X define the configuration space in which $X_{obs}$ is the obstacle region and where $X_{free}$ *=* X*/*$X_{obs}$ is the obstacle-free region. (X, $x_{start}$, $x_{goal}$) defines a path planning problem, where $x_{start}$∈ $X_{free}$ denotes the initial state and where $X_{goal}\in$ $X_{free}$ denotes the goal area. Let a continuous function σ: [0,1] → X of the bounded variation be path. Path σ is collision-free if ∀τ ∈ [0,1] and σ (τ) ∈ $X_{free}$.

Feasible path planning. For the path planning problem (X, $x_{start}$, $x_{goal}$), any solution corresponding to a feasible path σ such that σ is collision-free, σ (0) = $x_{start}$, and σ (1) = $x_{goal}$. If no solution exists, then a failure is reported.

Map Information Acquisition. Spatial data and obstacles are obtained through radar, sensors, or other devices, and input into the path planning algorithm.

Additionally, the relevant definitions of the concepts involved in the algorithm are as follows:

SampleFree: Sample point $x_{rand}$ is randomly selected from the map space. If $x_{rand}$ is within $X_{free}$, then $x_{rand}$. Otherwise, the sample is regenerated until the condition is satisfied.

CollisionFree: Given two points $x_{p1}$ and $x_{p2}$, check that the local path σ from $x_{p1}$ to $x_{p2}$ is collision-free on the map.

CollisionNode: Given two points $x_{p1}$ and $x_{p2}$, return the closest collision point $x_{p3}$ to $x_{p2}$ among the intersections of the path from $x_{p1}$ to $x_{p2}$ and the obstacle.

Create: Given three points, $x_{p1}$, $x_{p2}$, and $x_{p3}$. Create a node in the direction of $x_{p3}$ to $x_{p2}$ in terms of the length of the local path from $x_{p3}$ to $x_{p1}$, and return that node $x_{create}$.

Cost: Given point $x_{p}$ of *V*, it returns the full length of the path from $x_{start}$ to $x_{p}$.

Classify: Given point $x_{p}$ of *V*, return all nodes in $Near(x_{p})$ whose parent is not $Parent(x_{p})$.

Distance: Given two points $x_{p1}$ and $x_{p2}$, the Euclidean distance between $x_{p1}$ and $x_{p2}$.

Parent: Given point $x_{p}$ of *V*, it returns the parent vertex of $x_{p}$.

Nearest: Returns the point $x_{nearest}$ to $x_{rand}$ in the Euclidean distance.

Nears: Return the set of points $X_{near}$ contained in a hypersphere of a specific radius $R_{near}$ centered at $x_{rand}$.

InitialPathFound: Determines if an initial solution has been found.

### 2.2. F-RRT*

Algorithm 1 describes the execution process of the F-RRT*. The authors of F-RRT* reported that the parameter $R_{near}$ significantly affects the computation time for path planning. They also discovered that nodes close to obstacles could offer better paths.

To reduce the impact of $R_{near}$ on the computation time, F-RRT* omits the $R_{near}$ parameter during the ChooseParent phase. Instead, it selects a parent node for $x_{rand}$ in two steps: FindReachest (described in Algorithm 2) and CreateNode (described in Algorithm 3). FindReachest selects the reachable node $x_{reachest}$ from the ancestors of $x_{nearest}$. $x_{reachest}$ becomes the candidate parent node of $x_{rand}$. It is obvious that connecting $x_{reachest}$ and $x_{rand}$ makes the cost of the path from $x_{start}$ to $x_{rand}$ lower than connecting $x_{nearest}$ and $x_{rand}$. CreateNode then creates a new node $x_{create}$ via the dichotomy method, which can connect to both $x_{rand}$ and the parent node of $x_{reachest}$, thereby improving the quality of the path. During this process, the parameter $D_{dichotomy}$ serves as the terminating criterion for the dichotomy.

Although this method improves path quality, the frequent use of dichotomy in the CreateNode process leads to a high number of collision-detection operations, increasing the cost of creating new nodes. In addition, because F-RRT* does not recognize previously created nodes, many repeated operations are needed to create the same node.
Algorithm1 F-RRT***Require**$:\;x_{start},$ $X_{goal},\;Map,\;n_{max},\;R_{near},\;D_{dichotomy}$ **Ensure**: *G = (V, E)* 1: $V\;\leftarrow \;\{x_{start}\}$,E ← ∅ 2: for i=1$\;\mathrm{to}\;\;n_{max}$ do 3:······· $x_{rand}$← SampleFree(i) 4:·······$\;x_{nearest}\leftarrow \;Nearest(V,\;\;x_{rand})$ 5:········$if\;CollisionFree(x_{rand},\;x_{nearest})\;then$ 6:··············$X_{near}\leftarrow \;Nears(V,\;x_{rand},\;R_{near})$ 7:··············$x_{reachest}\leftarrow \;FindReachest(G,\;x_{nearest},\;x_{rand})$ 8:··············$x_{create}\leftarrow \;CreateNode(G,\;x_{reachest},\;x_{rand},\;D_{dichotomy})$ 9:··············$if\;x_{create}\;\neq \;\emptyset \;then$ 10:···················$V\;\leftarrow \;V\;\cup \;\{x_{create},\;x_{rand}\}$ 11:···················$E\;\leftarrow \;E\;\cup \;\{(Parent(x_{reachest}),\;x_{create}),\;(x_{create},\;x_{rand})\}$ 12:············else 13:···················*V ← V* ∪ *{*$x_{rand}$*}* 14:···················$E\;\leftarrow \;E\;\cup \;\{(x_{reachest},\;x_{rand})\}$ 15:············end if 16:············if InitialPathFound then 17: ···················Return G=(V, E) 18:·············end if 19:·······end if 20:·······$G\;\leftarrow \;Rewire(G,\;x_{rand},\;X_{near})$ 21: end for


**Algorithm 2 FindReachest**
**Require**$:\;G,\;x_{nearest},\;x_{rand},\;x_{start}$ **Ensure**: $x_{reachest}$ 1$:\;x_{reachest}\leftarrow \;x_{nearest}$ $2:\;\mathrm{while}\;x_{reachest}\neq x_{start}$ do 3:········$\mathrm{if}\;CollisionFree(x_{rand},\;Parent(x_{reachest}))\;$then 4:··············$x_{reachest}\leftarrow \;Parent(x_{reachest})$ 5:········else 6:···············$\mathrm{Return}\;x_{reachest}$ 7:········end if 8: end while 9: Return $x_{reachest}$



**Algorithm 3 CreateNode**
**Require**: $G,\;x_{nearest},\;x_{rand},\;D_{dichotomy}$ **Ensure**: $x_{create}$ 1$:\;x_{allow}\leftarrow \;x_{nearest}$ $2:\;\mathrm{if}\;x_{nearest}\neq x_{start}$ then 3:·········$x_{forbid}\leftarrow \;Parent(x_{nearest})$ 4:·········$\mathrm{while}\;Distance(x_{allow},\;x_{forbid})>D_{dichotomy}$ do 5:···············$xmid\;\leftarrow \;(x_{allow}+x_{forbid})/2$ 6:···············$\mathrm{if}\;CollisionFree(x_{rand},\;x_{mid})$ then 7:······················$x_{allow}\leftarrow \;x_{mid}$ 8:···············else 9:······················$x_{forbid}\leftarrow \;x_{mid}$ 10:·············end if 11:·······end while 12:·······$x_{forbid}\leftarrow \;x_{rand}$ 13: ······$\mathrm{while}\;Distance(x_{allow},\;x_{forbid})>D_{dichotomy}\;$do 14:··············$x_{mid}\leftarrow \;(x_{allow}+x_{forbid})/2$ 15:··············$\mathrm{if}\;CollisionFree(x_{mid},\;Parent(x_{nearest}))$ then 16:····················$x_{allow}\leftarrow \;x_{mid}$ 17:··············else 18:·····················$x_{forbid}\leftarrow \;x_{mid}$ 19:··············end if 20: ·······end while
21: end if

$22:\;\mathrm{if}\;\;x_{allow}\neq x_{nearest}$ then 23:········$x_{create}\leftarrow \;x_{allow}$ 24: else 25:········$x_{create}\leftarrow \;\emptyset$ 26: end if 27: Return $x_{create}$


### 2.3. MQ-RRT*

The MQ-RRT* algorithm introduces a new hybrid sampling strategy that combines a sparse sampling mechanism and a dynamic goal-biased mechanism. The sparse sampling mechanism effectively avoids repeated sampling within the same space by limiting the minimum distance between the random tree nodes. The goal-biased mechanism allows the algorithm to dynamically adjust the probability of selecting a goal node as a sample point on the basis of the growth speed of the random tree. Specifically, as the growth speed of the random tree increases, the probability of selecting the goal node as a sample point also increases, increasing the growth of the random tree to a certain degree of directionality, which helps accelerate the path search. In addition, MQ-RRT* introduces a new node creation method that improves the path quality of the algorithm in environments with circular obstacles.

## 3. Proposed Algorithm: FHQ-RRT*

**Figure 1:**
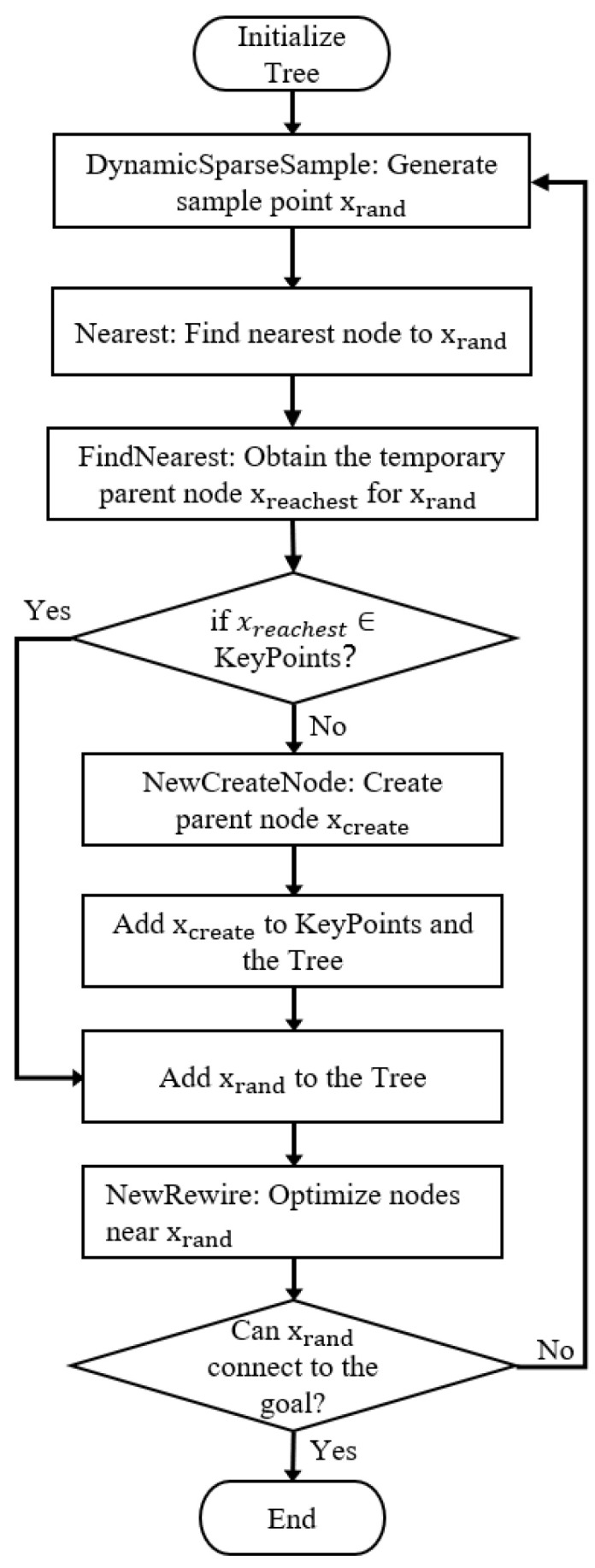
The flowchart of FHQ-RRT*.

In this section, we introduce the basic principles of the FHQ-RRT* algorithm. First, the algorithm proposes a dynamic sparse sampling strategy for the sampling phase that controls the density of the random tree node by dynamically adjusting the spacing between them, thereby achieving a balance between the exploration speed and accuracy of the algorithm. Second, the algorithm introduces a new node creation method and the concept of KeyPoints, aiming to reduce the cost and frequency of node creation while improving path quality. Finally, the algorithm presents a new rewiring strategy that focuses on the scope of the rewire in areas of higher value, thus improving the efficiency of rewiring. The pseudocode for the FHQ-RRT* algorithm is presented in Algorithm 4. Figure 1 illustrates the flowchart of FHQ-RRT*.
Algorithm 4 F HQ-RRT***Require**$:\;\;x_{start},\;x_{goal},\;Map,\;n_{max},\;\;R_{near},\;D_{dichotomy}{,\;\;\lambda }_{1},\;\;\lambda_{2},\;{\;D}_{1},\;{D\;}_{2}$ **Ensure**: G=(V,E) 1:$\;V\;\leftarrow \;\{x_{start}\;\},V_{keypoints}\;\leftarrow \;\{x_{start}\;\},E\;\leftarrow \;\emptyset$ 2: for $i=1\;to\;n_{max}\;$do 3:········$x_{rand}\;\leftarrow \;DynamicSparseSample(G,\;{\;\lambda }_{1},\;\;\lambda_{2},\;\;D_{1},\;{\;D}_{2})$ 4:········$x_{nearest}\leftarrow Nearest\left(G,x_{rand}\right)$ 4:········$x_{reachest}\leftarrow \;FindReachest(G,\;x_{nearest},\;x_{rand},\;\;x_{start})$ 4:········$\mathrm{if}\;\;x_{rand}$$,\;\;x_{reachest}$ is not None then 5:···············$\mathrm{if}\;\;x_{reachest}$$\;\mathrm{in}\;V_{keypoints}$ then 6:······················$V\;\leftarrow \;V\;\cup \;\{x_{rand}\;\}$ 7:······················$E\;\leftarrow \;E\;\cup \;\{(x_{reachest},\;\;x_{rand})\}$ 8:···············else 9:······················$x_{parent}=Parent(x_{reachest})$ 10:····················$x_{create}\;\leftarrow \;NewCreateNode(G,\;x_{parent},\;x_{reachest},\;\;x_{rand},\;\;D_{dichotomy})$ 11:····················$V\;\leftarrow \;V\;\cup \;\{x_{rand}\;\}$ 12:····················$E\;\leftarrow \;E\;\cup \;\{(x_{create},\;\;x_{rand})\}$ 13:············end if 14:············$\mathrm{if}\;CollisionFree(x_{rand},\;\;x_{goal})\;$then 15:····················Return G=(V,E) 16:············end if 17:·······end if 18:·······$G\;\leftarrow \;NewRewire(G,\;x_{rand},\;\;R_{near})$ 19: end for 20: Return G=(V,E)


### 3.1. Dynamic Sparse Sampling Strategy

The sparse sampling strategy of MQ-RRT* effectively avoids the repeated sampling of the explored space. However, owing to its limitation of the minimum distance between random tree nodes, this strategy lacks sufficient adaptability in response to environmental changes. In particular, maps with narrow spaces may fail to generate feasible paths. To overcome this limitation, this paper proposes a dynamic sparse sampling strategy that dynamically adjusts the distance between random tree nodes on the basis of the tree growth rate, thereby improving the adaptability of the algorithm to environmental changes. The core concept of the dynamic sparse sampling strategy is as follows.

When the growth speed of a random tree is fast, the environment is relatively simple. In this case, the algorithm selects a larger sparse distance, reducing the density of random tree nodes and thus accelerating the algorithm’s exploration speed.

When the growth speed of the random tree slows down, the environment becomes more complex. At this point, the algorithm selects a smaller sparse distance to increase the density of random tree nodes, thereby improving its ability to explore environmental details.

In extreme environments, especially those with narrow spaces, the algorithm completely eliminates the limitation of sparse distance and reverts to a random sampling strategy, allowing for the full release of the algorithm’s ability to explore environmental details.

The growth rate of a random tree ($\lambda_{rate})$ is defined as the reciprocal of the number of iterations ($n_{rate}$) required for the algorithm to successfully expand a new node. Its mathematical expression is as follows:

(1) $$\lambda_{\mathrm{rate}}=\frac{1}{n_{\mathrm{rate}}}$$

These operations are represented by a DynamicSparseSample function in Algorithm 5.
**Algorithm 5 DynamicSparseSample****Require**$:\;G{,\;\lambda }_{1},\;\lambda_{2},\;D_{1},\;D_{2}$ **Ensure**$:\;x_{rand}$ 1:$\;n_{rate}=1$ 2: for i=1 to n do 3:········$x_{rand}\;\leftarrow \;SampleFree()$ 4:········$x_{nearest}=Nearest(x_{rand})$ $5:\;\mathrm{if}\;\mathrm{not}\;CollisionFree(x_{nearest},\;\;x_{rand})\;\mathrm{then}$ $6:\;\;\;\;\;\;\;\;\;\;\;\;\;\;n_{rate}+1$ 7: Continue 8:········end if 9:········$\lambda_{rate}=1/n_{rate}$ 10:······$D_{sparse}=NearestNodeDistance(x_{nearest},\;{\;x}_{rand})$ 11:······$\mathrm{if}\;\lambda_{1}$$\;<\;\;\lambda_{rate}$ < 1 then 12:·············$\mathrm{if}\;D_{sparse}$$\;<\;D_{1}$ then 13:·····················$n_{rate}+1$ 14:·····················continue 15:·············end if 16:······$\mathrm{else}\;\mathrm{if}\;\lambda_{2}<\lambda_{rate}<\lambda_{1}\;$then 17:·············$\mathrm{if}\;D_{sparse}$ *<* $D_{2}$ then 18:·····················$n_{rate}+1$ 19:·····················continue 20:·············end if 21:······end if 22:······$\mathrm{Return}\;x_{rand}$ 23: end for 24: Return None


### 3.2. Improved Choose Parent Strategy

**Figure 2:**
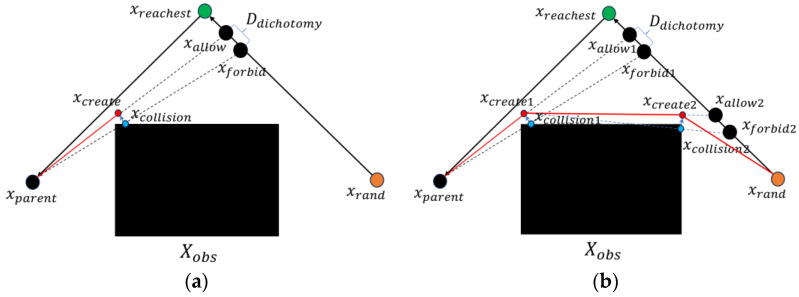
Principles of NewCreateNode and process for FHQ-RRT*. (**a**) Create a new node. (**b**) Recursively create nodes.

The processes of creating new nodes in both F-RRT* and MQ-RRT* are computationally expensive. To address these issues, this paper proposes a new node creation method called NewCreateNode, which reduces the cost of creating nodes. Algorithm 6 describes the execution process of NewCreateNode, the main principle of which is shown in Figure 2a, which uses a binary search to find a passable path and a collision path on the obstacle side. The CollisionNode process computes the collision point between the collision path and obstacle and returns the farthest collision point $x_{collision}$ from $x_{parent}$. The Create process then generates a new node $x_{create}$ on the passable path on the basis of the distance from $x_{collision}$ to $x_{parent}$.

Compared with the method used in F-RRT*, NewCreateNode reduces the cost of node creation by nearly half. Additionally, the new method supports recursive calls, allowing the algorithm to generate paths that are closer to obstacles. Figure 2b illustrates the effects of path generation through recursive calls.

The three algorithms mentioned in the background (F-RRT* and MQ-RRT*) frequently call the CreateNode process to create a new parent node whenever a new random sample point $x_{rand}$ is added, thereby replacing the candidate parent node for $x_{rand}$. This approach leads to the generation of many new parent nodes, especially at the corners of obstacles, where the new parent nodes are very close to each other and consume significant computational resources but actually contribute little to path optimization.

To address this issue, this paper introduced the concept of KeyPoints. The nodes created by the NewCreateNode process were marked as KeyPoints. When the algorithm detects that the candidate parent node of $x_{rand}$ belongs to KeyPoints, it directly adds $x_{rand}$ to the tree structure without creating a new parent node. This improvement aims to reduce unnecessary consumption of computational resources and enhance the efficiency of the algorithm.
**Algorithm 6 NewCreateNode****Require**$:G,\;x_{parent},\;x_{reachest},\;x_{rand},\;D_{dichotomy}$ **Ensure**: $x_{create}$ 1$:\;x_{allow}\;\leftarrow x_{reachest}$ $2:\;\mathrm{if}\;x_{reachest}\;\neq \;x_{start}$ then 3:········$x_{forbid}\;\leftarrow x_{rand}$ 4:········$\mathrm{while}\;Distance(x_{allow},\;\;x_{forbid})>\;D_{dichotomy}$ do 5:················$x_{mid}\;\leftarrow \;(x_{allow}+x_{forbid})/2$ 6:················$\mathrm{if}\;CollisionFree(x_{parent},\;\;x_{mid})$ then 7:························$x_{allow}\;\leftarrow \;x_{mid}$ 8:················else 9:························$x_{forbid}\;\leftarrow \;x_{mid}$ 10:·············end if 11:······end while 12:······$x_{collisionnode}\;\leftarrow \;CollsionNode(x_{parent},\;\;x_{forbid})$ 13:······$x_{create}\;\leftarrow \;Create(x_{collisionnode},\;x_{allow},\;x_{parent})$ 14:······$\mathrm{if}\;x_{create}$$\;\mathrm{not}\;\mathrm{in}\;V_{keypoints}$ then 15:············$V_{keypoints}$$\;\leftarrow \;V_{keypoints}$ ∪$\;\{x_{create}$ } 16:·············$V\;\leftarrow \;V\;\cup \;\{x_{create}\;\}$ 17:·············$E\;\leftarrow \;E\;\cup \;\{\;x_{parent},\;\;x_{create})\}$ 18:······end if 19:······$\mathrm{if}\;\mathrm{not}\;CollisionFree(x_{create},\;x_{rand})$ then 20:·············Return $NewCreateNode(G,\;x_{create},\;\;x_{allow},\;\;x_{rand},\;\;D_{dichotomy})$ 21:······end if 22: end if 23: Return $x_{reachest}$


### 3.3. Newrewire

**Figure 3:**
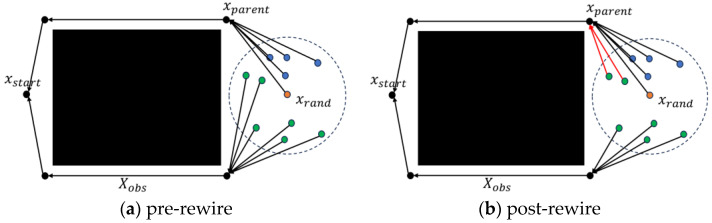
Principles of the NewRewire process for FHQ-RRT*(green dots: non-co-origin nodes; blue dots: co-origin nodes) (**a**) Before executing the NewRewire operation. (**b**) After executing the NewRewire operation.

In the Rewire phase, this paper proposes a new rewire strategy. Before rewiring the path, the algorithm introduces a Classification process to divide the nodes near $x_{rand}$ (denoted as $X_{near}$) into two categories: co-origin nodes and non-co-origin nodes. The co-origin nodes refer to the nodes that share the same parent as $x_{rand}$, whereas the non-co-origin nodes are the other nodes excluding the co-origin nodes, as shown in Figure 3a. This classification aims to prevent the algorithm from processing co-origin nodes, as they share the same parent with $x_{rand}$, and processing them does not improve path quality. On the other hand, focusing on the rewiring operation on non-co-origin nodes can effectively improve the efficiency of path rewiring.

In addition, a path-rewire strategy for non-co-origin nodes was introduced. Specifically, for any node $x_{sibling}$ in the set of non-co-origins, if it is connected to the parent node of $x_{rand}$ and the path cost of $x_{sibling}$ is reduced, then the parent node of $x_{sibling}$ is updated to the parent node of $x_{rand}$. This improvement improved the quality of the path, as shown in Figure 3b. These operations are represented via the NewRewire function in Algorithm 7.
**Algorithm 7 NewRewire****Require**$:G,\;x_{rand},\;R_{near}$ **Ensure**: G=(E,V) 1$:\;X_{siblings}\leftarrow \;Classify(x_{rand},\;R_{near})$ 2$:\;x_{parent}\;\leftarrow \;Parent(x_{rand})$ $3:\;\mathrm{for}\;\mathrm{each}\;x_{sibling}\;\in \;X_{siblings}$ do 4:·······$\mathrm{if}\;Cost(x_{sibling})>Cost(x_{parent})+Distance(x_{parent},\;x_{sibling})\;$then 5:··············$\mathrm{if}\;CollisionFree(x_{parent},\;x_{sibling})$ then 6:·····················$E\;\leftarrow \;(E\backslash \{(Parent(x_{sibling}),\;x_{sibling})\})\;\cup \;\{(x_{parent},\;x_{sibling})\}$ 7:··············end if 8:·······end if 9: end for 10: Return G=(V,E)


## 4. Experimentation

**Figure 4:**
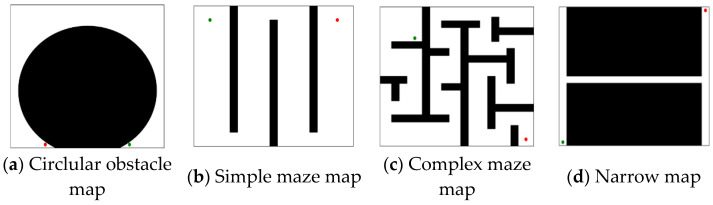
The simulation maps (the green dot: $x_{s}$, the red dot: $x_{g}$).

In this section, we conduct simulations of the FHQ-RRT* algorithm and compare its performance with those of three other algorithms—RRT*, F-RRT*, and MQ-RRT*—in four 2D map environments: the circular obstacle map, simple maze map, complex maze map, and narrow map. Each map was 200 × 200 units in size; the simulation environments are shown in Figure 4.

RRT* was chosen as the baseline algorithm, and FHQ-RRT* was compared with MQ-RRT* and F-RRT* because these two algorithms are all path planning algorithms based on triangle inequality, with MQ-RRT* being the latest representative of this class of algorithms. To minimize the effect of randomness, each algorithm was run 100 times during the simulation, and the experimental data were averaged to ensure the reliability of the comparison results.

The basic performance of the four algorithms was evaluated via the following three fundamental indicators:

$T_{i}$: Represents the time required for algorithm i to generate a feasible path.

$N_{i}$: Represents the total number of nodes in the random tree when algorithm i generates a feasible path.

$C_{i}$: Represents the cost of the feasible path generated by algorithm i.

Additionally, to show the effective improvement brought by the enhancements proposed in FHQ-RRT*, we introduce four additional indicators for comparison (it is important to note that RRT* is not involved in the comparison of these additional indicators because it is not an optimization algorithm based on the triangle inequality).

${FC}_{i}$: Represents the frequency with which algorithm i invokes the CreateNode process. This frequency is measured as the ratio of the total number of times the CreateNode process is called during the generation of feasible paths to the total number of iterations performed by algorithm i. This parameter reflects the impact of the KeyPoints concept on the performance of the algorithm.

${CC}_{i}$: Represents the cost of executing the CreateNode process in algorithm i. Specifically, it is measured by the number of collision checks required during the execution of the CreateNode process.

${RC}_{i}$: Represents the cost of rewiring the path via the *Rewire* process in algorithm i. This cost is measured by the number of nodes that must be rewired during the execution of the Rewire process. Because the number of nodes processed in the Rewire process is related to the density of the random tree nodes, ${RC}_{i}$ indirectly reflects the impact of the sparse sampling mechanism on the algorithm’s performance.

${RC}_{new}$: To demonstrate the advantages of the proposed NewRewire process, we define ${RC}_{new}$ as the execution cost of the NewRewire process in FHQ-RRT*.

The algorithm terminates when either a feasible path is found or the maximum number of iterations is reached. All the simulations were performed on a computer equipped with a Core i7-12800X CPU and 16 GB of RAM.

### 4.1. Parameter Selection

In the simulation process, the following parameters need to be identified: the query radius $R_{near}$, maximum iteration number $n_{max}$, binary search parameter $D_{dichotomy}$, and parameters related to the dynamic sparse sampling strategy: $\lambda_{1},\lambda_{2},D_{1},D_{2}$. The parameter of the sparse sampling distance for MQ-RRT* is $D_{MQ}$.

Hu et al. [33] demonstrated in their paper that as $D_{dichotomy}$ increases, the path cost rises while the time cost decreases. A larger $R_{near}$ will lead to an increase in the number of nodes processed during the rewiring phase, thereby increasing the time cost. The parameters $D_{1}$ and $D_{2}$ control the sparsity of the sampling points, directly affecting the algorithm’s exploration efficiency. In simple obstacle maps, larger values of $D_{1}$ and $D_{2}$ can accelerate the exploration speed. However, in complex obstacle maps, excessively large $D_{1}$ and $D_{2}$ values can increase the sampling failure rate, thereby slowing down the path planning speed. Therefore, it is advisable to set smaller $D_{1}$ and larger $D_{2}$ values to achieve rapid exploration in simple obstacle maps and reduce the sampling failure rate while maintaining a certain exploration speed in complex obstacle maps. The parameters $\lambda_{1}$ and $\lambda_{2}$ influence the growth rate of the random tree and its sensitivity to environmental complexity. Larger $\lambda_{1}$ and $\lambda_{2}$ values can enhance the algorithm’s sensitivity to complex environments, enabling it to adjust the sparse distance $D_{1}$ or abandon the sparsity constraint earlier, thereby ensuring successful path planning. However, excessively large λ values can make the algorithm overly sensitive, slowing down the exploration of the environment and increasing the planning time. Overall, it is recommended to select smaller $\lambda_{1}$ and $D_{1}$ values and larger $\lambda_{2}$ and $D_{2}$ values to balance the algorithm’s performance across different environments.

A larger $R_{near}$ and a smaller $D_{dichotomy}$ increase the algorithm’s computation time but can yield more optimal paths. Therefore, we set $R_{near}$ = 25 and $D_{dichotomy}$ = 1. To accommodate the environmental complexity in the four maps, we set $\lambda_{1}$ = 0.2, $\lambda_{2}$ = 0.1, $D_{1}$ = 10, and $D_{2}$ = 15. Additionally, we set $D_{MQ}$ = 10 and $n_{max}$ = 10000.

### 4.2. Circular Obstacles Map

**Figure 5:**
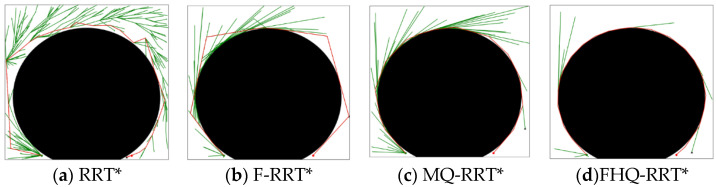
Performance of algorithms in a circular obstacle map (the green dot: $x_{s}$, the red dot: $x_{g}$).

Figure 5 shows the simulation results of the four algorithms in the circular obstacle map, where the red line represents the feasible path. As shown in the figure, both MQ-RRT* and FHQ-RRT* generate shorter and smoother paths. This demonstrates that, like MQ-RRT*, FHQ-RRT* has the ability to generate more optimal paths around arc-shaped obstacles. This is mainly due to the recursive calls of NewCreateNode, which enable the algorithm to generate a series of nodes that closely follow the obstacles’ surfaces. Compared with MQ-RRT*, FHQ-RRT* reduces the path cost by 1.2%. As can be seen from the data in Table 1, the nodes generated by NewCreateNode in FHQ-RRT* are closer to the obstacles, thereby reducing the path cost.

In terms of time cost $T_{i}$, FHQ-RRT* reduces it by 40% compared with other algorithms, mainly due to the synergistic optimization of the dynamic sparse sampling strategy, key point identification, and rewiring mechanism. In the circular obstacle map, the random tree grows rapidly. FHQ-RRT* dynamically adjusts the sparse sampling to select a larger sparse distance, reducing the node density and accelerating the exploration of the environment. The $N_{i}$ value of FHQ-RRT* is 39.5% lower than that of MQ-RRT*, which also proves the effectiveness of the dynamic sparse sampling strategy in improving the algorithm’s performance. In addition, key point identification reduces the frequency of calling the node creation program, lowering the computational overhead. According to the data in Table 2, the $R_{new}$ indicator shows that the number of nodes that FHQ-RRT*’s NewRewire process needs to handle is 86% less than that of other algorithms’ Rewire process. This is because the NewRewire process does not need to process co-origin nodes. Moreover, the ${CC}_{i}$ indicator shows that the execution cost of FHQ-RRT*’s NewCreateNode is 50.1% lower than that of other algorithms. This further proves the effectiveness of the relevant improvements in the node creation program.

**Figure 6:**
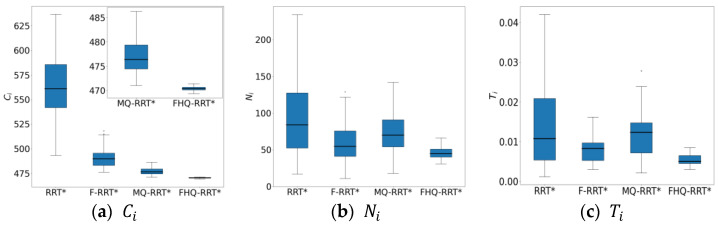
The statistical results of $C_{i},\;N_{i},\;T_{i}$ in the circular obstacle map.

Figure 6 shows the box plots of the basic indicator data for the four algorithms, further demonstrating FHQ-RRT*’s advantage in data stability and indicating better consistency in its performance.

### 4.3. Simple Maze Map

**Figure 7:**
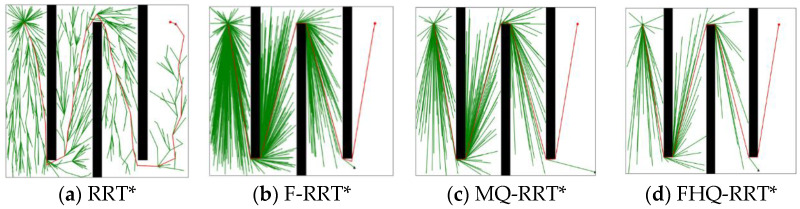
Performance of algorithms in simple maze map (the green dot: $x_{s}$, the red dot: $x_{g}$).

Figure 7 shows the simulation results of the four algorithms in the simple maze map. Except for RRT*, the path costs generated by the other algorithms are very close, as their planned paths are near-optimal. However, according to the data in Table 3, the path cost indicator $C_{i}$ shows that FHQ-RRT* still achieves a lower path cost than the other algorithms, primarily due to its generation of nodes closer to obstacles, which further reduces the path cost in path planning.

In terms of time cost $T_{i}$, FHQ-RRT* reduces it by 77% compared with other algorithms. This is because, in the simple maze map, the rapid growth of the random tree prompts FHQ-RRT*’s dynamic sparse sampling strategy to select a larger sparse distance, thereby reducing the sampling density and accelerating the exploration of the environment. In comparison of the number of nodes $N_{i}$, the FHQ-RRT* algorithm reduces them by 55.5% relative to other algorithms, thereby further substantiating the efficacy of the dynamic sparse sampling strategy in reducing the number of nodes. Additionally, according to the data in Table 4, the $R_{new}$ indicator shows that FHQ-RRT*’s NewRewire process reduces the number of processed nodes by 96.5% by focusing the rewiring operation on more valuable areas, thereby reducing computational overhead. Meanwhile, FHQ-RRT* reduces computational cost by identifying key points to avoid frequent calls to the node creation process. The combined effect of these improvements gives FHQ-RRT* a significant advantage in terms of time cost compared with other algorithms.

**Figure 8:**
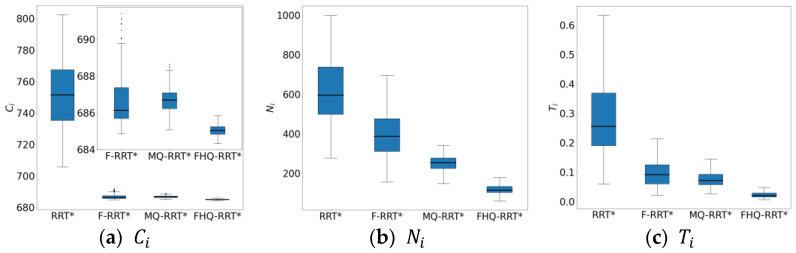
The statistical results of $C_{i},\;N_{i},\;T_{i}$ in the simple maze map.

The box plots of the basic indicator data for FHQ-RRT* in Figure 8 further confirm its superiority in terms of data stability, thereby verifying its excellent performance in path planning.

### 4.4. Complex Maze Map

**Figure 9:**
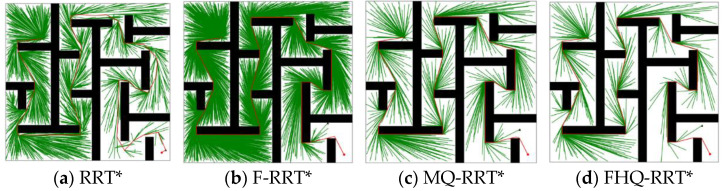
Performance of algorithms in complex maze map (the green dot: $x_{s}$, the red dot: $x_{g}$).

Figure 9 shows the simulation results of the four algorithms in the complex maze map. According to the data in Table 5, except for RRT*, the paths generated by the other algorithms are close to the optimal solution, leading to closely matched $C_{i}$ values. However, the nodes created by the NewCreateNode process of FHQ-RRT* are closer to the obstacles, thereby achieving the lowest path cost.

FHQ-RRT* and MQ-RRT* both employ sparse sampling strategies to reduce node density, resulting in significantly lower $N_{i}$ compared with F-RRT* and RRT*. The $N_{i}$ value of FHQ-RRT* is 30% lower than that of MQ-RRT*, as the slower tree growth rate in the complex maze map causes FHQ-RRT*’s dynamic sparse sampling strategy to select smaller sparse distances more frequently. However, when the tree growth rate increases, it switches to larger sparse distances, thereby further reducing the number and density of nodes.

In terms of time cost, FHQ-RRT* reduces it by 56.6%, 90.8%, and 91.8% compared with MQ-RRT*, F-RRT*, and RRT*, respectively. This advantage is mainly attributed to two factors: First, the dynamic sparse sampling strategy accelerates the algorithm’s exploration of the environment, thereby reducing time overhead. Second, according to the data in Table 6, the low computational cost and low invocation frequency of NewCreateNode, along with the improved rewiring efficiency of the NewRewire process due to fewer processed nodes, collectively reduce the computation time.

**Figure 10:**
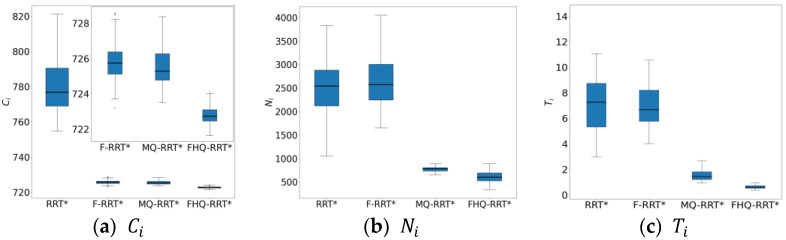
The statistical results of $C_{i},\;N_{i},\;{and\;T}_{i}$ in the complex maze map.

The box plots of the basic indicators for the four algorithms in Figure 10 further confirm the advantage of FHQ-RRT* in terms of data stability.

### 4.5. Narrow Map

During the experiments, the narrower the gap in tight spaces was, the higher the failure rate of the MQ-RRT* algorithm. However, the FHQ-RRT* algorithm, which employs a dynamic sparse sampling strategy, did not encounter this issue. Specifically, when the navigable gap is excessively narrow, the dynamic sparse sampling strategy of FHQ-RRT* abandons the sparse distance constraint and reverts to random sampling, thereby ensuring the successful acquisition of a feasible path. To ensure that the sparse sampling strategy of MQ-RRT* could obtain sampling points and complete the planning task, the width of the narrow gap was set to 10 units.

**Figure 11:**
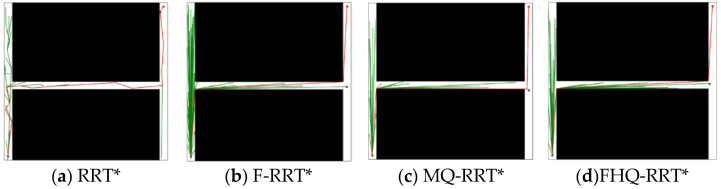
Performance of algorithms in narrow map (the green dot: $x_{s}$, the red dot: $x_{g}$).

Figure 11 shows the simulation results of the four algorithms in the narrow map environment. According to the data in Table 7, except for RRT*, the paths planned by the other three algorithms are close to the optimal solution, resulting in similar $C_{i}$ values. However, due to the closer proximity of nodes created by the NewCreateNode process of FHQ-RRT* to the obstacles, the $C_{i}$ value of FHQ-RRT* remains the lowest.

In terms of node count $N_{i}$, the sparse sampling strategy, which limits node density, results in lower node counts for FHQ-RRT* and MQ-RRT* compared with the other two algorithms. Regarding time cost $T_{i}$, F-RRT* reduces it by 42.8% compared with MQ-RRT*, as the sparse distance constraint in MQ-RRT* lowers the sampling success rate in the narrow map, thereby increasing the algorithm’s runtime. Although FHQ-RRT* is also subject to the sparse distance constraint, according to the data in Table 8, its low computational cost and low invocation frequency of the NewCreateNode process, along with the improved rewiring efficiency of the NewRewire process due to fewer processed nodes, significantly reduce the runtime, making it 50% lower than that of F-RRT*.

**Figure 12:**
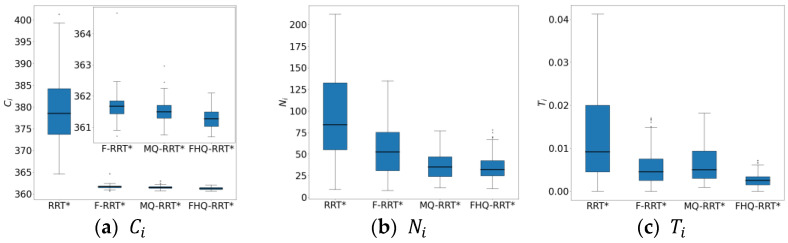
The statistical results of $C_{i},\;N_{i},\;and\;T_{i}$ in a narrow map.

Figure 12 shows the box plots of the basic indicator data for the four algorithms, further demonstrating FHQ-RRT*’s advantage in data stability and indicating better consistency in its performance.

## 5. Results Analysis

These experimental results indicate that the FHQ-RRT* algorithm outperforms the other four algorithms. The main reasons for this can be summarized as follows:

1. Dynamic Sparse Sampling Strategy: The FHQ-RRT* algorithm uses a dynamic sparse sampling strategy that adjusts the sparse distance on the basis of the growth rate of the random tree, balancing the exploration speed and accuracy of the algorithm. This strategy improves the adaptability of the algorithm to the environment, particularly in narrow-space environments.
2. Reduced Node Creation Cost: Through the NewCreateNode process, the algorithm reduces the cost of creating new nodes. In addition, the introduction of the KeyPoints concept reduces the frequency of calling in the NewCreateNode process. These two improvements reduce the overall cost of the algorithm for node generation.
3. Enhanced Rewire Efficiency: The new Rewire focuses on rewiring operations in areas with relatively high values, reducing the number of nodes that need to be processed and thus improving the algorithm performance efficiency.

## 6. Discussion

This paper presents a sampling-based path planning algorithm, FHQ-RRT*, that can obtain high-quality initial solutions more quickly. The main contributions of FHQ-RRT* can be summarized as follows. First, it introduces a dynamic sparse sampling strategy that allows the algorithm to balance exploration speed and accuracy on the basis of the growth rate of the random tree, enhancing the adaptability of the algorithm. Second, a new node creation strategy is proposed, along with the introduction of the KeyPoints concept. This strategy reduces the cost of creating nodes while providing smoother paths, and the introduction of the KeyPoints concept decreases the frequency of node creation. Third, a new *Rewire* strategy is proposed that focuses on the scope of the rewire in areas of higher value, thus reducing the number of nodes that need to be processed. The superiority of FHQ-RRT* in terms of performance was verified through simulation experiments on four maps and comparisons with other algorithms.

Although FHQ-RRT* performs well, its practical application requires integration with the kinematic model of the robot, and future research will focus on this aspect.

## Figures and Tables

**Table 1:** The average value of $C_{i},\;N_{i},\;T_{i}$ in the circular obstacle map.

Algorithm *i*	$\mathrm{Average}C_{i}$	$\mathrm{Average}N_{i}$	$\mathrm{Average}T_{i}$
RRT*	560.37	94.05	0.013
F-RRT*	488.98	61.16	0.007
MQ-RRT*	476.07	74.52	0.011
FHQ-RRT*	470.38	45.06	0.005

**Table 2:** The average values of ${RC}_{i}$*,* ${RC}_{new}$*,* ${CC}_{i}$*,* and ${FC}_{i}$ in the circular obstacle map.

Algorithm *i*	$\mathrm{Average}\;{RC}_{i}({RC}_{new})$	$\mathrm{Average}\;{CC}_{i}$	$\mathrm{Average}\;{FC}_{i}$
F-RRT*	2.71(-)	7.24	97.6%
MQ-RRT*	2.33 (-)	9.21	96.8%
FHQ-RRT*	1.28(0.32)	3.61	51.1%

**Table 3:** The average value of $C_{i},\;N_{i},\;T_{i}$ in the simple maze map.

Algorithm *i*	$\mathrm{Average}\;C_{i}$	$\mathrm{Average}\;N_{i}$	$\mathrm{Average}\;T_{i}$
RRT*	757.11	611.44	0.301
F-RRT*	686.76	398.52	0.095
MQ-RRT*	686.73	256.67	0.083
FHQ-RRT*	685.07	114.08	0.019

**Table 4:** The average values of ${RC}_{i}$*,* ${RC}_{new}$*,* ${CC}_{i}$*, and* ${FC}_{i}$ in the simple maze map.

Algorithm *i*	$\mathrm{Average}\;{RC}_{i}({RC}_{New})$	$\mathrm{Average}\;{CC}_{i}$	$\mathrm{Average}\;{FC}_{i}$
F-RRT*	14.49 (-)	9.98	99.7%
MQ-RRT*	8.02 (-)	5.77	99.5%
FHQ-RRT*	3.18 (0.28)	4.82	6.1%

**Table 5:** The average value of $C_{i},\;N_{i},\;and\;T_{i}$ in the complex maze map.

Algorithm *i*	$\mathrm{Average}C_{i}$	$\mathrm{Average}N_{i}$	$\mathrm{Average}T_{i}$
RRT*	779.17	2412.96	7.539
F-RRT*	725.77	2603.33	6.694
MQ-RRT*	725.34	766.72	1.415
FHQ-RRT*	722.81	625.04	0.614

**Table 6:** The average values of ${RC}_{i}$*,* ${RC}_{new}$*,* ${CC}_{i}$*,* and ${FC}_{i}$ in the complex maze map.

Algorithm *i*	$\mathrm{Average}{RC}_{i}({RC}_{new})$	$\mathrm{Average}{CC}_{i}$	$\mathrm{Average}\;{FC}_{i}$
F-RRT*	75.63 (-)	8.76	99.95%
MQ-RRT*	17.93 (-)	5.42	99.79%
FHQ-RRT*	17.81(4.81)	4.27	8.32%

**Table 7:** The average value of $C_{i},\;N_{i},\;and\;T_{i}$ in a narrow map.

Algorithm *i*	$\mathrm{Average}\;C_{i}$	$\mathrm{Average}\;N_{i}$	$\mathrm{Average}\;T_{i}$
RRT*	377.78	98.33	0.013
F-RRT*	361.74	45.37	0.004
MQ-RRT*	361.56	37.12	0.007
FHQ-RRT*	361.32	35.28	0.002

**Table 8:** The average values of ${RC}_{i}$, ${RC}_{new}$, ${CC}_{i}$, and ${FC}_{i}$ in the narrow map.

Algorithm *i*	$\mathrm{Average}{RC}_{i}({RC}_{new})$	$\mathrm{Average}{CC}_{i}$	$\mathrm{Average}\;{FC}_{i}$
F-RRT*	4.02 (-)	12.47	97.4%
MQ-RRT*	3.57 (-)	12.67	95.6%
FHQ-RRT*	3.16 (0.05)	5.97	5.4%

## Data Availability

The original contributions presented in the study are included in the article. Further inquiries can be directed to the corresponding author.

## Abbreviations

The following abbreviations are used in this manuscript:

D*	Dynamic A*
